# The Use of a Radial Basis Function Neural Network and Fuzzy Modelling in the Assessment of Surface Roughness in the MDF Milling Process

**DOI:** 10.3390/ma16155292

**Published:** 2023-07-27

**Authors:** Krzysztof Szwajka, Joanna Zielińska-Szwajka, Tomasz Trzepieciński

**Affiliations:** 1 Department of Integrated Design and Tribology Systems, Faculty of Mechanics and Technology, Rzeszow University of Technology, ul. Kwiatkowskiego 4, 37-450 Stalowa Wola, Poland; 2 Department of Component Manufacturing and Production Organization, Faculty of Mechanics and Technology, Rzeszow University of Technology, ul. Kwiatkowskiego 4, 37-450 Stalowa Wola, Poland; j.zielinska@prz.edu.pl; 3 Department of Manufacturing Processes and Production Engineering, Rzeszow University of Technology, al. Powstańców Warszawy 8, 35-959 Rzeszów, Poland; tomtrz@prz.edu.pl

**Keywords:** MDF, medium-density fiberboard, milling, surface roughness

## Abstract

Wood-based composites are increasingly used in the industry not only because of the shortage of solid wood, but above all because of the better properties, such as high strength and aesthetic appearance compared to wood. Medium-density fiberboard (MDF) is a wood-based composite that is widely used in the furniture industry. In this work, an attempt was made to predict the surface roughness of the machined MDF in the milling process based on acceleration signals from an industrial piezoelectric sensor installed in the cutting zone. The surface roughness parameter Sq was adopted for the evaluation and measurement of surface roughness. The surface roughness prediction was performed using a radial basis function (RBF) artificial neural network (ANN) and a Takagi–Sugeno––Kang (TSK) fuzzy model with subtractive clustering. In the research, as inputs to the ANNs and fuzzy model, the kinematic parameters of the cutting process and selected measures of the acceleration signal were adopted. At the output, the values of the surface roughness parameter Sq were obtained. The results of the experiments show that the surface roughness is influenced not only by the kinematic parameters of the cutting, but also by the vibrations generated during the milling process. Therefore, by combining information on the cutting kinematics parameters and vibration, the accuracy of the surface roughness prediction in the milling process of MDF can be improved. The use of TSK fuzzy modelling based on the subtractive clustering method for integrating the information from many acceleration signal measurements in the examined range of cutting conditions meant the surface roughness was predicted with high accuracy and high reliability. With the help of two tested artificial intelligence tools, it is possible to estimate the surface roughness of the workpiece with only a small error. When using a radial neural network, the root mean square error for estimating the value of the Sq parameter was 0.379 μm, while the estimation error based on fuzzy logic was 0.198 μm. The surface of the sample made with the cutting parameters v_c_ = 76 m/min and v_f_ = 1200 mm/min is characterized by a less concentrated distribution of ordinate densities, compared to the surface of the sample cut with lower feed rates but at the same cutting speed. The most concentrated distribution of ordinate density (for the cutting speed v_c_ = 76 m/min) is characterized by the surface, where the feed rate value was v_f_ = 200 mm/min, with 90% of the material concentrated in the profile height of 28.2 μm. When using an RBF neural network, the RMSE of estimating the value of the Sq parameter was 0.379 μm, while the estimation error based on fuzzy logic was 0.198 μm.

## 1. Introduction

Medium-density fiberboard (MDF) is widely used for displays, signs, shop fronts, facades, and fittings [1]. MDF is produced from lignified fibers with or without the addition of a binder, and also with the addition of fillers [2]. Most fiberboard is produced as multilayered, with the layers differing in chemical composition, additives and degree of fragmentation [3].

The use of MDF in the industry is associated with machining when producing furniture. The milling process is one of the most commonly used operations in the production of MDF-based furniture. The machinability of MDF is determined by the quality of the machined surface. Penman et al. [4] developed a prototype inspection system for wood panels to detect the defects at production rates. The prototype uses a combination of pipelined processing modules and general purpose processor detect textural variation and color of MDF panels. Lin et al. [5] analyzed the appearance of MDF machines surface by passing a cutting tool through it at a relatively low speed. A digital camera was used that travels synchronously with the tool and the machinability characteristics of the MDF were detected. 

A number of studies report about the effect of cutting speed and feed rate on the surface roughness after milling MDF. Aquilera [6], Deus et al. [7] and Gaitonde et al. [8] observed a tendency of decreasing mean roughness (Ra parameter) with increasing cutting speed or decreasing feed rates. On the other hand, it was observed that the Ra-value increased with the reduction in the cutting speed. Davim et al. [9] studied the effect of various cutting parameters on the MDF surface roughness in the milling process using uncoated carbide cutting tools. They found that the surface roughness increased with feed rate and decreases with an increase in the tool rotational speed. Bal et al. [10] found that the surface roughness of milled MDF increased with the stepover and feed rate. As the feed rate increased, the resulting surface quality of the MDF declined [11]. Gaitonde et al. [8] used the Taguchi approach for optimization of the surface roughness in the milling of MDF. It was found that the surface roughness can be optimized with a higher cutting speed and lower feed rate values. Based on the analysis of variance, Ayyildiz [12] found the most effective parameter affecting the surface roughness is the depth of cut while the factor ranked second is cutting the speed. Li et al. [13] investigated the effects of input parameters on specific cutting energy during the MDF helical up-milling process. It was found that, although the surface roughness parameters Ra and Rz increased approximately 58.3% and 46.2%, respectively, under the optimal milling parameters, the optimization was feasible at the initial rough machining stage. 

Smart machining is an area of research where researchers are exploring various sensing methods and data processing to monitor machining processes online. Machining processes are affected by various factors related to the workpiece, tool and cutting parameters. Since it is difficult to assess the complex relationships between input and output factors using conventional quality control, developing an online system using advanced detection technologies seems to be a major goal for intelligent machining. Among the many commercially available sensors for monitoring machining processes, the most commonly used are force, vibration, acceleration and acoustic emission sensors. These sensors are often used to monitor processes such as turning [14], drilling [15], milling [16] or grinding [17]. However, taking into account the extreme conditions in the cutting zone during the processing of wood-based materials, there is a high probability of sensor damage due to the prevalence of very high amounts of dust. For this reason, cheaper sensors are used there, that is, acceleration sensors.

Wojciechowski et al. [18] investigated the ploughing phenomena in the tool flank face–workpiece interface during ball-end milling of AISI L6 alloy steel The authors developed original model of ploughing forces involved the effect of ploughing volume and minimum uncut chip thickness. It was concluded that progressing tool wear and surface inclination angle strongly effect on the edge forces which monotonic increase with growth of tool wear. Jarosz et al. [19] analyzed the effect of variable radial depth of cut on cutting force values in face milling processes of AW-6061-T6 aluminum alloy. With the proposed optimization strategy reduced the machining time for the analyzed face milling operation approximately 37% without exceeding imposed process parameter constraints. Chuchała et al. [20] investigated the surface roughness of AW-6086-T6 aluminum alloy thin plates manufactured with the cold rolling (CR) process after face milling process. The Authors proposed the milling strategy which take into account the depth of the material with included residual stresses after cold rolling. It was found that the CR direction of milled AW-6086-T6 plates does not affect the roughness of face-milled surfaces. Furthermore, the thickness of the workpiece did not significantly affect the surface roughness of machined surfaces. Pimenow et al. [21] reviewed the direct and indirect methods of tool condition monitoring in milling operations of metallic materials. The advantages, disadvantages, and prospects of using such sensors for milling operations are discussed in this review article. Artificial intelligence and conventional sensory systems were considered. The advantages and disadvantages of using sensory systems for milling are also discussed. Wojciechowski et al. [22] proposed an original analytic–experimental approach for predicting cutting forces during micro-end milling of AISI 1045 carbon steel considering the geometric errors of the machine tool–cutting tool system kinematics of micro-end milling and elastic-plastic deformations of the workpiece correlated with the chip thickness accumulation. It was found that the instantaneous force oscillations as a function of feed per tooth is related to the multiple cutting mechanism transitions observed during micro-milling in the burnishing-dominant regime. 

In recent years, there has been a strong development of research related to the use of fuzzy systems and artificial intelligence in decision-making processes. One of the possible applications of fuzzy systems to support decision making is the use in the production control process. Artificial intelligence methods, that is, fuzzy logic [23], approximate clustering via the mountain method [24], fuzzy-net based modelling [25], artificial neural networks (ANNs) [26], have been used more and more often, and intelligent machining monitoring is currently developing dynamically. Fuzzy systems are characterized by the method of knowledge representation, which is presented in a symbolic way by means of fuzzy rules and processing. Takagi and Sugeno [27] and Sugeno and Kang [28] have proposed an alternative reasoning system based on the rules of a special format, which is characterized by function-type consequents. In the conclusions of the rules, function-type consequents appear not as fuzzy sets, but as functions of input variables, and these are usually linear functions, so each rule of the model describes one flat (linear) segment of the model surface. In the rule base, functional dependence is used in the conclusions. It means that in the Takagi–Sugeno model there are no explicit output membership functions and sharpening functions.

The optimal determination of the parameters of CNC machining of MDF is extremely important, especially in finishing processes such as painting, varnishing or wrapping, where a specific surface roughness is required [29]. Many rejected parts occurring in the final stage of finishing MDF have their causes already at the initial stage of machining wood-based boards (cutting, milling, drilling). This is mainly due to improperly selected cutting parameters. Properly selected parameters are the most important factors increasing the quality of the machined surface [30]. Determining the optimal values of CNC parameters, which contribute to improving the quality of the machined surface, is necessary for a properly carried out process of milling materials for the furniture industry. Many scientific works are focused on the determination of the influence of cutting parameters on the surface roughness of MDF in the machining process. However, the optimal parameters in these studies were generally found using the Taguchi method and statistical methods [31]. Artificial neural networks have found wide application in wood science, e.g., in recognizing wood species [32], wood-drying process [33], predicting some mechanical properties of wood [34], optimizing process parameters in the manufacturing process of wood products [35], classification of wood and veneer defects [36], calculation of thermal conductivity of wood [37], analysis of moisture in wood [38] and prediction of wood cracking resistance [39]. Although there are many studies on the impact of machining parameters on wood surface roughness, there is a lack of research on modeling the impact of these parameters.

Advanced signal processing techniques and artificial intelligence play a key role in the development of modern tool condition monitoring systems. The most frequently chosen methods are neural networks [40], Mamdani fuzzy logic [41], a combination of a neural network with Mamdani fuzzy logic [42] or a genetic algorithm [43]. Fuzzy logic [44] provides a simple way to arrive at a final conclusion based on unclear, ambiguous, imprecise or missing input information. However, most of the published research works on this topic concern only metalworking. In the field of processing wood materials, there are no such works or there are very few of them, and they concern the use of neural networks. The aim of this article was to propose a fuzzy logic-based approach to surface roughness modeling based on the acceleration signal in the milling process of wood-based materials. For this purpose, subtractive clustering was additionally applied to the learning process. Subtractive clustering was used to partition the input space and extract a set of fuzzy rules. The least squares algorithm was used to determine the optimal membership functions together with the resulting rule base parameters. The obtained results were compared with the results obtained using radial neural networks. 

In this article, the surface roughness of the machined MDF in milling process is predicted based on acceleration signals from an industrial piezoelectric sensor installed in the cutting zone. Prediction of the surface roughness parameter Sq was performed using a radial basis function (RBF) ANN and a Takagi–Sugeno–Kang (TSK) fuzzy model with subtractive clustering. The kinematic parameters of the cutting process and selected measures of the acceleration signal were selected as input parameters. At the output, the value of the surface roughness parameter Sq was predicted.

## 2. Experimental Procedure

### 2.1. The Workpiece and the Cutting Tool

In the tests, a commercial MDF board with a thickness of 18 mm was used as the workpiece. The mechanical and physical properties of the processed material are shown in Table 1.

**Figure 1:**
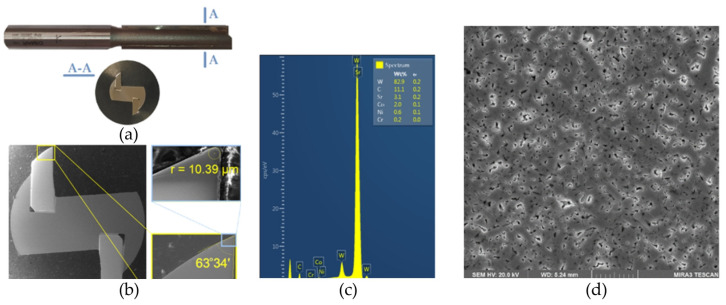
(**a**) The cutting tool, (**b**) the blade geometry, (**c**) the results of the spectral analysis, and (**d**) the microstructure of the blade material.

**Figure 2:**
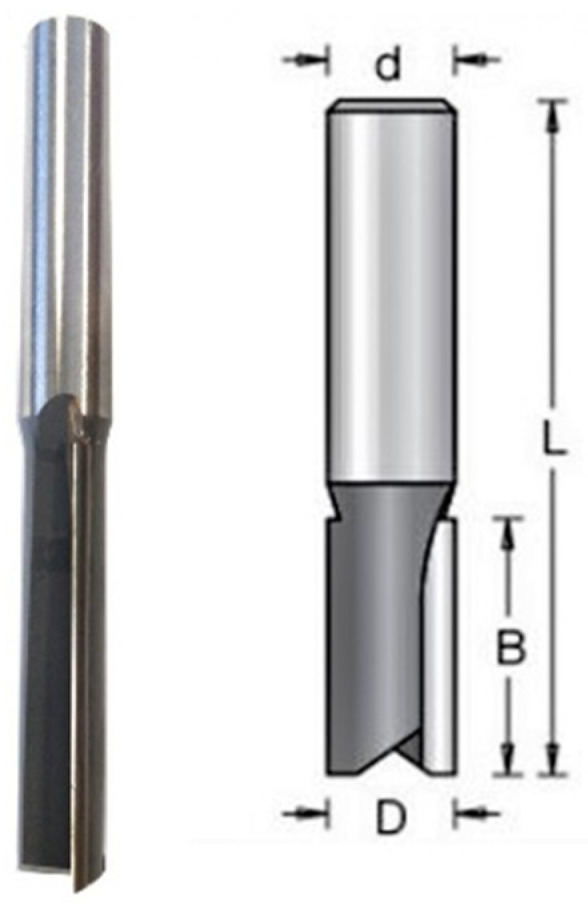
Geometry of the cutting tool.

A commercial cutting tool designed for machining MDF, a 12 mm diameter double-edged shank cutter HM 12 × 51 with sintered carbide tips (Figure 1a) produced by Dimar (Warsaw, Poland), was used in the research. The geometry and dimensions of the cutting tool are specified in Figure 2 and Table 2, respectively. Before starting the tests, a milling cutter (as used in the tests) was cut on an electrical discharge machining (machine. A metallographic specimen was prepared, which was used for spectral analysis and for measuring the basic geometry of the cutting tool blade. A spectral analysis of the chemical elements in the sintered carbide (Figure 1c) and analysis of its microstructure (Figure 1d) were carried out using an MIRA3 scanning electron microscope (TESCAN, Brno, Czech Republic). The blade angle and the radius of the rounding of the cutting edge of the blade (Figure 1b) were also measured.

### 2.2. Equipment and Machining Conditions

**Figure 3:**
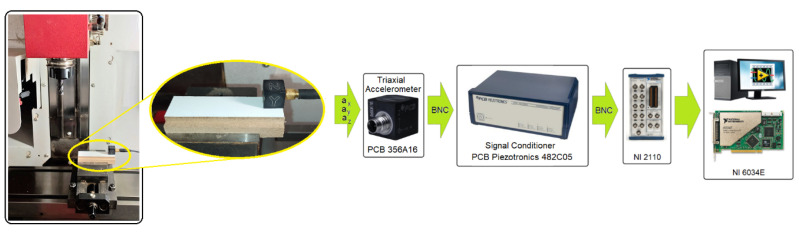
Experimental set-up and schematic of the data acquisition system.

The milling process was carried out on an EMCO^®^ CNC vertical milling machine (EMCO GmbH, Hallein, Austria). A schematic diagram of the configuration of the measurement track and the measurement data archiving system is shown in Figure 3. As part of the tests, acceleration signals in the directions a_x_, a_y_ and a_z_ coming from the cutting zone during the machining of the MDF were recorded on the CNC milling machine.

**Figure 4:**
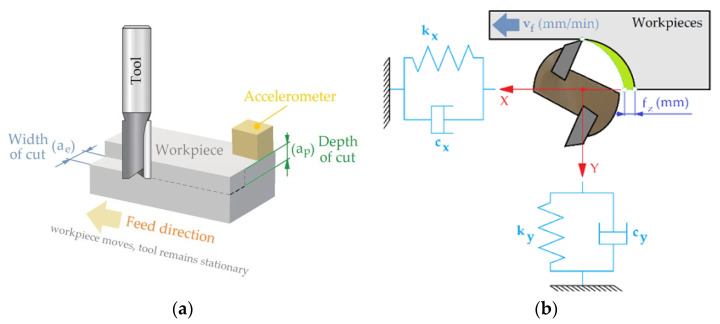
(**a**) The geometric parameters of the milling process; (**b**) the dynamics of the milling process.

As part of the tests, a surface with dimensions of 130 × 30 × 18 mm was milled using an end mill. The value of the acceleration signal in three mutually perpendicular directions was measured using a piezoelectric acceleration sensor PCB^®^ 356A16 (PCB PIEZOTRONICS, Depew, NY, USA) mounted on the workpiece (Figure 4a). The dynamics of the milling process are shown in Figure 4b.

**Figure 5:**
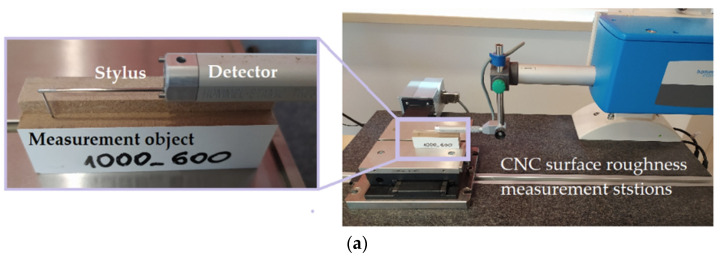

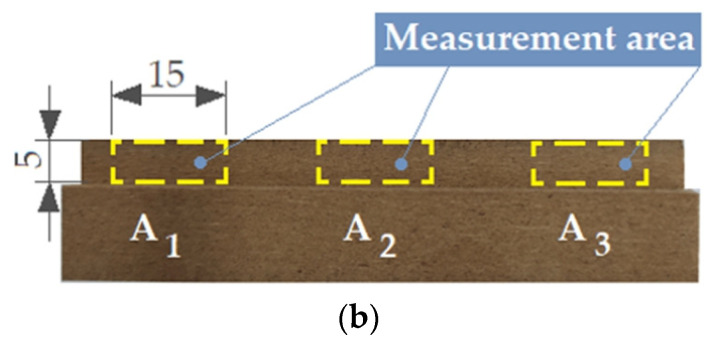
(**a**) Measurement of the surface topography and (**b**) the measurement area.

Signals from the sensor were recorded on a personal computer disc in digital form via a National Instruments^®^ 6034E (Austin, TX, USA) analogue-to-digital card. The sampling frequency of the signals during the experiments was 50 kHz, and the measurement resolution of the card was 16 bits. For each milling pass, the surface topography was measured using a CNC profilometer Hommel-Etamic T8000RC (Jenoptik, Jena, Germany). The surface topography was measured in three places on sections 15 mm long and 5 mm wide of the machined surface (at the milling depth a_p_) (Figure 5). 

The Sq is a statistical parameter with relatively low sensitivity to measurement errors and is therefore often used in surface measurements. Therefore, this parameter was used to characterize the surface roughness of machined surfaces. S_q_ parameter is the root mean square height of the surface. This parameter is defined as the root mean square value of the surface departures *z*(*x*,*y*), within the sampling area:

(1) $$S_{q}=\sqrt{\frac{1}{A}\iint_{A}^{}z^{2}(x,y)dxdy}$$

where A is the sampling area, z is the surface height position, and x, y are lengths in perpendicular directions.

The surface topography measurement methodology is described in detail in [2].

Table 3 summarizes the cutting parameters used during the milling experiments. Three repetitions were made for each set of cutting parameters. Each operation consisted of 18 treatments, the parameters of which are presented in Table 3. The tests were carried out in parallel using two identical milling cutters. The operations carried out with the first tool and the second tool are denoted as *Net_1* and *Net_2*, respectively.

### 2.3. System Dynamic Characteristics

An important issue in the analysis of the acceleration (vibration) signal in the frequency domain is to determine the dynamic characteristics of the mass-dissipation-elastic (MDE) system (Figure 4b). This can be obtained by mathematical modelling or experiments. A real MDE system has many degrees of freedom (shapes and modal values of vibration) in many directions. For most problems, it is sufficient to assume two perpendicular directions for the MDE system susceptibility (Figure 4b). To determine the dynamic characteristics of the MDE system, an input function was applied to the input of the system. Such an input function is the Dirac impulse:

(2) $$\delta \left(t\right)=\left\{\begin{array}{cc} 0\; & t\neq 0\\ +\infty \; & t=0 \end{array}\right.$$

**Figure 6:**
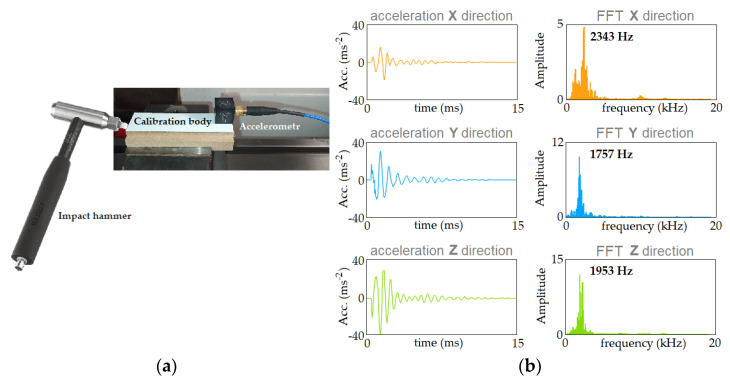
(**a**) The impulse excitation and (**b**) the time waveforms and their spectra extracted from the whole signal.

Technically, the Dirac impulse is replaced here by a short-term excitation, a blow with a hammer equipped with a force sensor. A KISTLER 9724A (Kistler, Winterthur, Switzerland) modal hammer (Figure 6a) was used in the tests. As part of the analysis, a modal analysis was made from the measured signal, containing signals of excitation force and acceleration of the object, enabling determination of the dynamic characteristics of the MDE system. An important advantage of impulse excitation is that the impact energy distribution is visible in the continuous spectrum with regard to the frequency which is approximately the inverse of the impulse duration.

Using the LabVIEW environment, a program was developed to analyze the dynamic characteristics of the system. It allows for automatic analysis of the recorded signals in terms of their usefulness to determine the dynamic characteristics of the MDE system. Manually reviewing the entire signal, evaluating every hit and response and rejecting incorrect ones requires a lot of work and time, as well as experience and attention. The automatic fragmentation used in the program is based on the detection of the beginning of a single hit. It is recognized based on the force signal—crossing a threshold that is five times the maximum force value from the first 100 signal samples. Then, 750 samples were extracted. For the sampling frequency used of 50 kHz, the single hit window is 15 ms. A longer window may cause successive hits to overlap, which the program would recognize as an invalid hit. Figure 6b shows an example of the acceleration (Acc) and Fast Fourier Transform (FFT) variations, extracted from the entire signal using the above mentioned criteria.

The analyses allowed determination of the natural frequencies of the MDE system in three analyzed directions: ω_ox_ = 2343 Hz; ω_oy_ = 1757 Hz and ω_oz_ = 1953 Hz. It is known that the frequency of cutting the successive cutter blades is [4]:

(3) $$\omega_{f_{z}}=\frac{n*z}{60}$$

where n—tool rotational speed (rpm); z—number of milling cutter teeth.

This means that multiplicity of the crossing frequency of the blades should be different from the natural frequency in order to avoid the resonant frequency. This leads directly to forcing vibrations at the resonance frequency, which is very close to the natural frequency:

(4) $$\omega_{rez}=\omega_{o}\sqrt{1-2d^{2}}$$

For most machine tools, the damping factor *d* is very small (0.001–0.05), so $\sqrt{1-2d^{2}}\approx 1$.

### 2.4. Data Collection and Analysis

**Figure 7:**
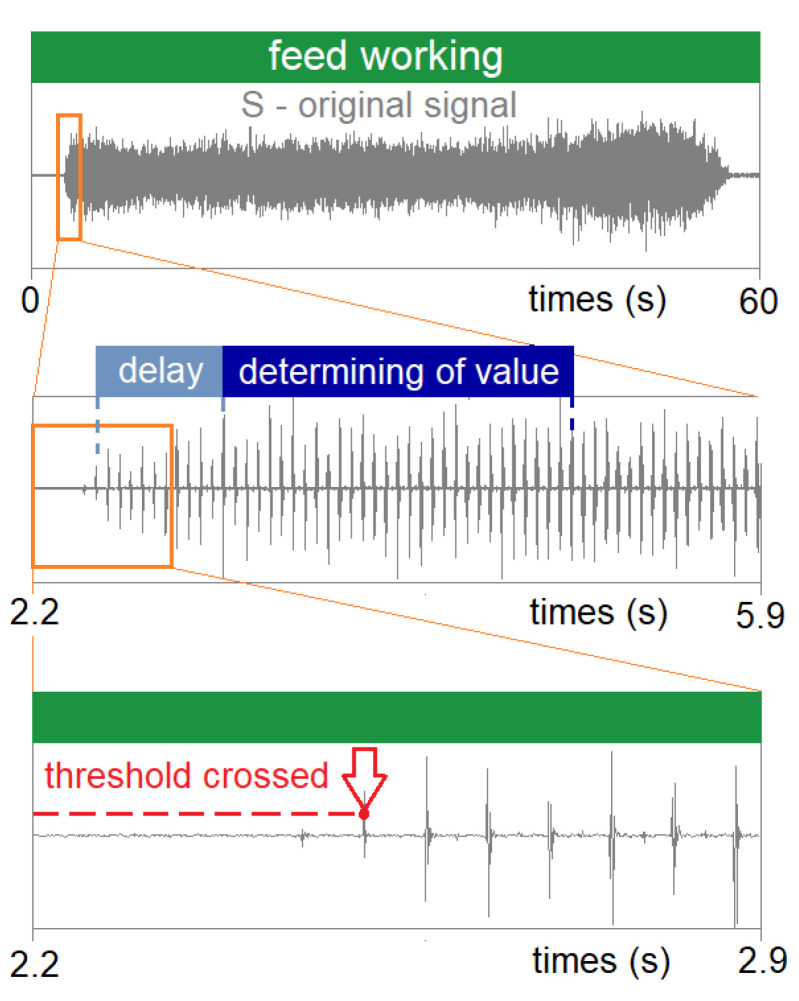
Methodology for determining the signal measurements.

For the analysis of the recorded acceleration signals, a computer program (in the LabVIEW environment) was created, enabling the determination of selected measurement values of the recorded signals in selected time intervals (Figure 7). The operation of the program consists of the automatic determination of the values of the registered signals in strictly defined time intervals.

**Figure 8:**
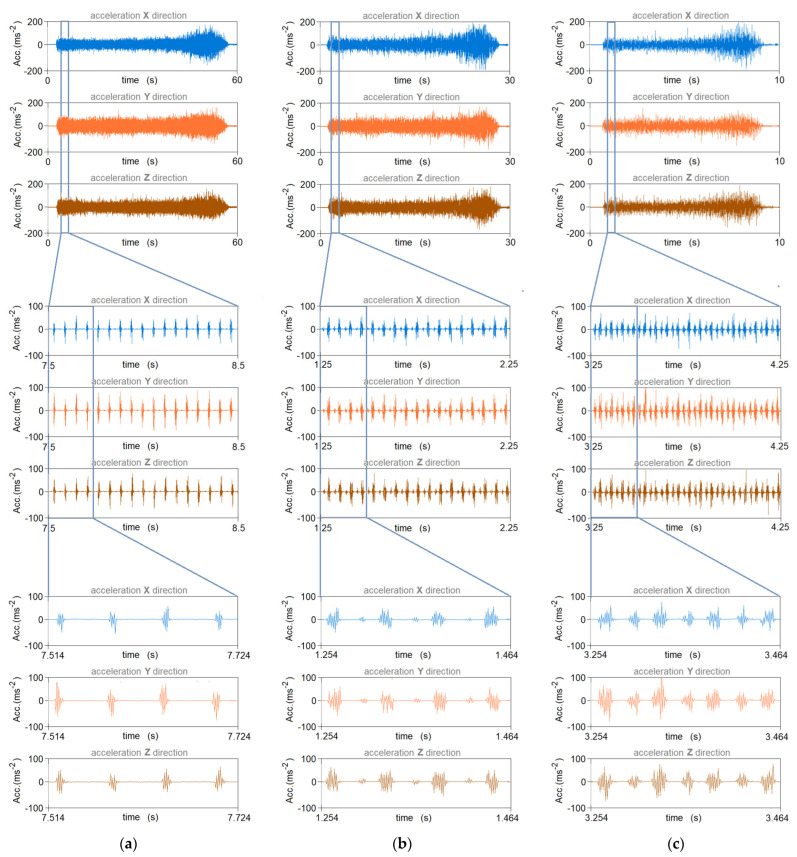
Accelerometer (Acc) signal in the MDF milling process for the following parameters: (**a**) v_c_ = 114 m/min, f_z_ = 0.15 mm; (**b**) v_c_ = 76 m/min, f_z_ = 0.15 mm; (**c**) v_c_ = 38 m/min, f_z_ = 0.15 mm.

A detailed description of the program is given in [1]. Figure 8 shows the changes in acceleration signals for the directions a_x_, a_y_ and a_z_, depending on the value of the feed rate (v_f_) and the cutting speed (v_c_).

**Figure 9:**
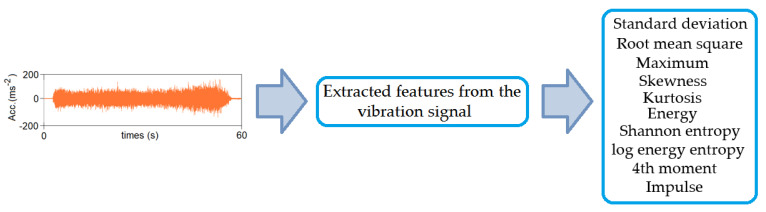
Extracted features from the vibration signal.

To determine the measures of the recorded acceleration signals in the directions a_x_, a_y_ and a_z_ in the time and frequency domain, it was decided to adopt commonly known features (Figure 9) used in the analysis of surface roughness evaluation. Mathematical expressions for their determination are described in [45].

## 3. Results and Discussion

### 3.1. Surface Topography

Nowadays, three-dimensional (3D) topographic surface analysis is gradually gaining more and more popularity in the industry, and it seems that its role in design and production will become more and more important in the future. Surface topography is understood as a set of detailed three-dimensional features of a certain limited area of surface. The 3D roughness measurements are used to better understand the nature of the surface topography. All issues related to the cooperation of two surfaces are three-dimensional phenomena, therefore descriptions cannot be limited to two-dimensional analysis. The 3D measurements refer to the surface and are referred to as topography measurements, stereometry or stereometric measurements, while 2D measurements refer to the profile and are referred to as profile roughness measurements. Both 2D and 3D measurements are referred to as roughness measurements, which is a simplification, because roughness is further defined as a set of the smallest surface irregularities with relatively small spacing, usually including irregularities that are the result of the specificity of the production process, excluding waviness and shape error [46].

**Figure 10:**
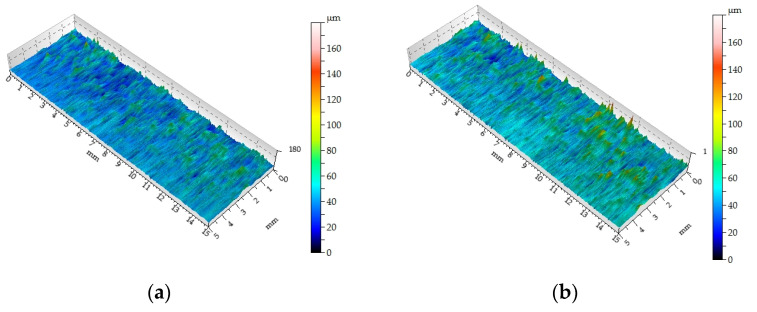

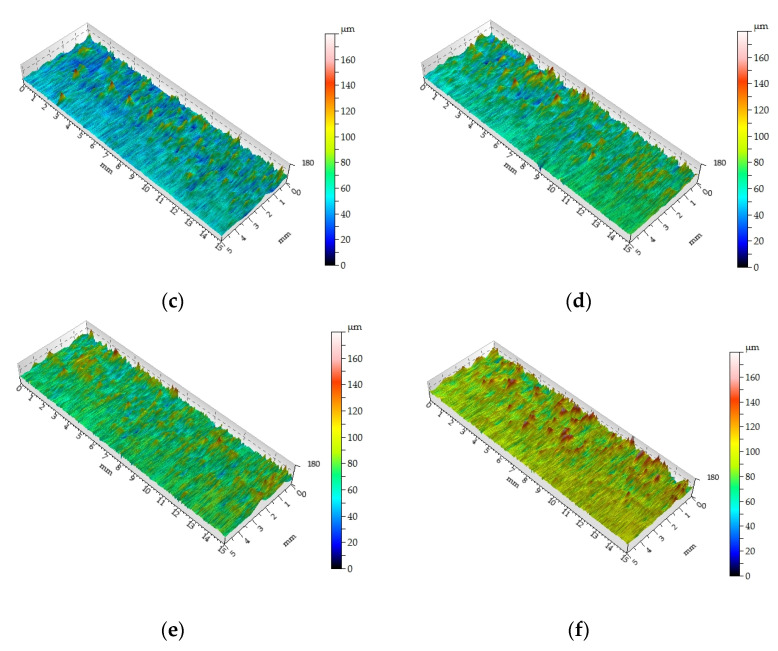
Surface topography of the milled surface for following parameters: (**a**) cutting speed v_c_ = 76 m/min and feed rate v_f_ = 200 mm/min; (**b**) v_c_ = 76 m/min and v_f_ = 400 mm/min; (**c**) v_c_ = 76 m/min and v_f_ = 600 mm/min; (**d**) v_c_ = 76 m/min and v_f_ = 800 mm/min; (**e**) v_c_ = 76 m/min and v_f_ = 1000 mm/min; (**f**) v_c_ = 76 m/min and v_f_ = 1200 mm/min.

Figure 10 shows the surface topographies obtained in the milling process at a cutting speed of 76 m/min for six feed rates. On the basis of the topography map, in selected measurement sections, the Sq parameter was determined.

In the presented surface topography maps, two areas of different surface topography can be clearly observed. The first area occurs in the outer layers of the MDF, at a depth of approximately 3 mm. This differentiation can be explained by the multilayered construction of the MDF. Szwajka et al. [1] presented the distribution of density and hardness on the cross-section of the MDF. The resulting surface topography accurately reflects changes in both the hardness and density of the board material [47].

In the conducted research on the surface roughness of the machined surface, the relationships generally known from the literature [6,9] and from the authors’ earlier research were confirmed regarding the impact of both the cutting speed and the feed rate on the surface roughness of the machined surface. A decreasing trend for the Sq parameter was observed with increasing cutting speed and/or decreasing feed rate.

Another analyzed feature of the topography was the Abbott–Firestone curve, which describes the distribution of material in the profile [48]. It should be treated as a percentage increase in the share of individual topography points for the entire analyzed area. From a mathematical point of view, this graph can be treated as a distribution of the probability of finding a point in a given area with a height lower than for the given coordinates. On this basis, it is possible to find the properties of a given profile in terms of the utility functions of the geometric structure of the surface. The horizontal axis of the Abbott–Firestone curve represents the load bearing coefficient in percentage terms, while the vertical axis represents the depth determined in the measurement units [49].

**Figure 11:**
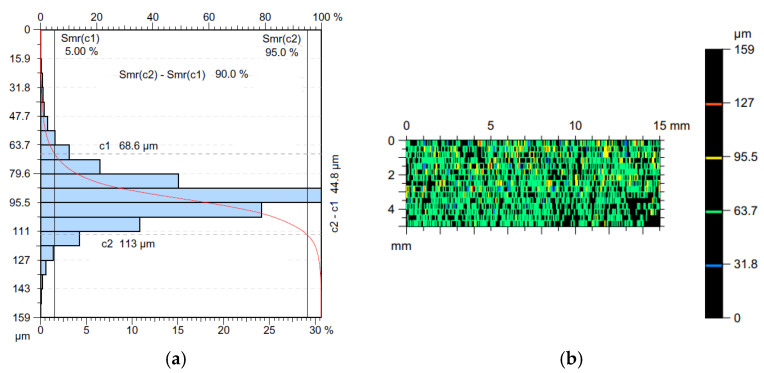
(**a**) The Abbott–Firestone curve and (**b**) the contour plot of the surface obtained for the cutting parameters v_c_ = 76 m/min and v_f_ = 1200 mm/min.

Figure 11 shows the material ratio curve of the surface obtained in the milling process for the following cutting parameters: v_c_ = 76 m/min, v_f_ = 1200 mm/min. The surface of the sample made with the cutting parameters described above is characterized by a less concentrated distribution of ordinate densities, compared to the surface of the sample cut with lower feed rates but at the same cutting speed. For the surface shown in Figure 10f, 90% of the material is concentrated in the 44.8 μm range (Figure 11a). The most concentrated distribution (for the cutting speed v_c_ = 76 m/min) is characterized by the surface where the feed rate value was v_f_ = 200 mm/min, with 90% of the material concentrated in the profile height of 28.2 μm. For surfaces machined with the parameters of v_c_ = 76 m/min and v_f_ = 1200 mm/min, more than 28% of the material is concentrated at a profile height of approximately 90 μm, while for the surface machined with a feed rate value v_f_ = 200 mm/min, more than 31% of the material is concentrated at a profile height of approximately 73 μm.

The results show that the tool rotational speed and feed rate can affect the surface roughness and the resulting value of the Sq parameter in the MDF milling process. Finding the optimal combination of v_c_ and v_f_ can help achieve the desired surface roughness [8,50]. A contour plot (Figure 11b) is a basic tool in the analysis of surface roughness and topography according to ISO 25178.

With lower v_c_ values and higher v_f_ values we can get a rougher surface texture with more peaks and valleys. On the other hand, higher v_c_ and higher v_f_ can lead to a more significant material removal rate, resulting in a smoother surface texture. However, higher v_c_ and lower v_f_ can lead to higher cutting temperatures, which have an adverse effect on the MDF, which can result in a rougher surface texture.

### 3.2. Application of the Neural Network to Evaluate the Surface Roughness

Radial basis networks available in the Matlab^®^ Neural Network Toolbox ver. R2018a (Natick, MA, USA) were used to map the input data to the surface roughness parameter Sq. During numerous experiments with RBF-type networks [51,52], their limited usefulness for the evaluation of some specific cases was discovered. The propagation function of the input neurons of this network is radial, in the MATLAB environment it is the Gaussian function, and the network divides the input data subspace into areas that are in the zone of influence of subsequent neurons. Such a network poorly approximates data beyond the range on which it was trained. This is connected with the basic disadvantage, that is, the requirement to plan and conduct more extensive studies with more measurements. It is necessary to sufficiently cover the entire range of variability of the cutting parameters. This is quite cumbersome and is a major disadvantage of RBF neural networks compared to feed forward back propagation (FFBP) networks. Dozens of experiments with RBF neural networks were carried out. First, we checked how the RBF network responds to different sets of input data. The only way to influence the predicted result of the RBF network is to set the parameterized shape of the radial function of each input neuron. The higher the value of the spread parameter, denoted as *s_p_*, the more flattened the radial function is. This required additional attempts to optimize the *s_p_* parameter.

In order to determine the optimal value of the mentioned parameter *s_p_* using the empirical method, numerous network tests should be carried out on data from all trials. It can be calculated using the analytical method based on a complicated mathematical apparatus. However, that was not the purpose of this work. Based on our own experience and series of attempts, we can assume *s_p_* = 30,000. The response of the RBF network for low values of this parameter (*s_p_* = 500) coincides with the real values of the Sq parameter. This is consistent with the networking algorithm, which is based on the assumption that the number of radial neurons is equal to the number of training vectors [53]. Since the areas with a smaller radius in the input data space covered by neurons with a lower *s_p_* overlap only in the immediate vicinity, they do not significantly affect the neurons further away from them. Thanks to this, it is possible to accurately approximate the training data with splined radial functions with a smaller *s_p_* value. For a large value of *s_p_*, approximation errors are obtained, calculated by the MATLAB program, which are the result of the overlapping of a larger number of Gaussian surfaces. As in the case of the FFBP networks, the goal is not to get a perfect result for the training data, but to equalize the errors for all the data sets. The optimal value of the *s_p_* coefficient is obtained as a result of a compromise between the minimization of errors of the training set and the ability to generalize for the testing set. In further tests with this type of network, the value *s_p_* = 30,000 was assumed.

A comparison of the response quality of the networks trained on various combinations of input data was made in order to analyze the usefulness of the measurements and cutting parameters in terms of their use in predicting the surface roughness value using the RBF network. A number of sets with different combinations of variables were taken into account (Table 4). The lack of usefulness of some measurements of the acceleration signals for assessing the surface roughness of the machined surface was also verified here. The most favorable result is marked in bold in Table 4. The accuracy of estimation of the surface roughness parameter Sq is evaluated based on the root mean square error (RMSE):

(5) $$RMSE=\sqrt{\sum {\left({Sq}_{\left(r\right)}-{Sq}_{\left(e\right)}\right)}^{2}/N}$$

where Sq_(r)_ and Sq_(e)_ are the measured and predicted Sq-values, respectively; N is number of measurements.

The selection of data for training the RBF network was deeply justified and well thought out. When training the FFBP network, a balance between the training and testing errors is searched for by influencing the training process through weight disturbances and the pruning of neurons [53,54]. If relying only on information about training errors, it would be difficult to adopt the criterion of stopping the network training process. For RBF networks, training on a selected set of data has a similar meaning. In addition, as mentioned earlier, RBF networks have a weaker ability to extrapolate and interpolate results than FFBP networks. This means, and is confirmed in the research, that the training samples must cover the entire range of input data variability.

**Figure 12:**
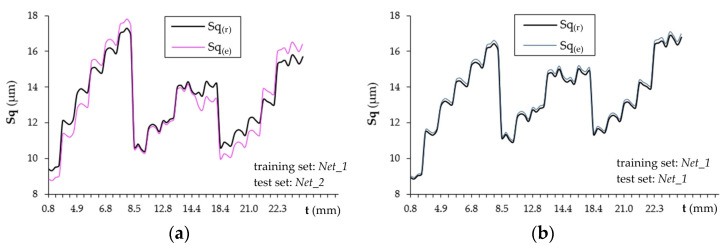
Responses of RBF networks tested on the (**a**) *Net_2* and (**b**) *Net_1* sets.

Figure 12a presents the responses of the network tested on the *Net_2* set and trained on the *Net_1* set based on the cutting speed v_c_, feed rate v_f_ and selected measurements of the acceleration signals (Table 4). Figure 12b shows the responses of the network trained and tested on the *Net_1* set.

The graphs shown in Figure 12 confirm the previous conclusions drawn from the analysis of network training and testing. In the *Net_1* set, the initial Sq values were at a higher level than in the *Net_2* set. This is reflected in the assessment of the Sq parameter by the RBF network. A characteristic decrease or increase in the value of the Sq parameter at the output of the RBF network proves that the values of input variables have exceeded the training range and it is a serious disadvantage of this type of network [55]. Changes in the Sq parameter throughout the milling duration were shown on purpose to indicate large fluctuations in the network response and poor approximation of the data corresponding to the value of the Sq parameter (Figure 12a).

### 3.3. Subtractive Clustering-Based TSK Fuzzy Modelling

One of the methods of optimizing the fuzzy model parameters are clustering methods. They use the fact that automatic detection of certain groups of measurement points and characteristic patterns of system behavior can be represented by one rule or their coherent set. The mountain clustering method developed by Yager and Filev [24] is one of the best methods for dividing a given set into a certain number of clusters/subsets. However, in order to create specific subsets, it is necessary to establish the so-called cluster measures [56]. In general, the center of such a cluster can be any point in the measured space. However, this approach leads to computationally expensive algorithms with exponential complexity. The subtractive potential method created by Chiu [57] and described in this article goes in a slightly different direction. It assumes that the center of the cluster can only be an element of the set of measurement points. The Chiu method [57] therefore determines the beginnings of clusters composed of a single point, which is the starting point of the searched subset. In the case of the subtractive potential method, the searched space is therefore limited to a separate set of points.

Cluster centers determined using the subtractive potential method can be used to build fuzzy reasoning rules for various artificial intelligence algorithms [46,58]. In particular, they can be used to build models that predict the behavior of various types of complex systems over time—in other words, to create machine learning algorithms.

System identification using clustering involves creating clusters in the data space and translating these clusters into TSK rules so that the model obtained is similar to the identified system. The purpose of the subtractive clustering identification algorithm is to estimate both the number and initial location of cluster centers and to extract the TSK fuzzy rules from the input/output data [56]. Subtractive clustering works by finding an optimal data point to define a cluster center based on the density of the surrounding data points [58]. This method is a fast-clustering method for solving large dimension problems with a moderate number of data points. This is because the calculation requirements in this method grow linearly with the increase in the dimension of the data and the square of the number of data points. Classical logic does not provide adequate tools for the analysis of complex systems, where goals and input-output dependencies are often imprecisely defined, and thus difficult to quantify. Hence, there has been significant progress in the application of methods based on fuzzy logic. Its techniques are based on reasoning similar to human reasoning, and therefore have a wide spectrum of practical applications, especially in modelling and control issues. The idea of subtractive mountain clustering consists of determining each point xi and the function P representing the potential of this point. Let us consider a set of N data points {x_1_, x_2_, …, x_n_} defined by m-dimensional x_j_. In order not to lose the value of the data, this function should be taken as a normalized space, so all data is normalized to the interval [0, 1]. It is therefore assumed that the potential at the i-th point of the set is expressed by the following formula:

(6) $$P\left(i\right)=\sum_{j=1}^{N}e_{i}^{-\alpha {\left\|x_{i}-x_{j}\right\|}^{2}}$$

for i = 1, …, N, and α = 4/(r_a_)^2^ for a certain constant r_a_ > 0. It is clear from the form of the potential function that the potential of a set point is higher the more other points are in its immediate vicinity. This property makes the subtractive potential method much more resistant to disturbances caused by random points than clustering algorithms such as C-means. After assigning the potential P(i) to each point of the set, the first center of the cluster is selected, which is always the point with the greatest potential. Therefore, c_1_ = x_u_, where u = arg_i_ max P(i), and P(u) is denoted by P* and we consider it a reference potential for the selection procedure of the remaining cluster measurements. In addition, when we select the center of the next cluster c_k_ = x_u_ (for the correct u) we modify the value of the potential function assigned to individual points of the set as follows:

(7) $$P\left(i\right)=P\left(i\right)-P(u)e^{-\beta {\left\|x_{i}-x_{j}\right\|}^{2}}$$

where β = 4/(r_b_)^2^ for some r_b_ > 0 which is a constant defining the range of the potential function. For practical reasons, it is commonly assumed that r_b_ > r_a_ and the most common value is r_b_ = 1.25r_a_. The selection of subsequent cluster centers is made until the potential of all points exceeds a certain fixed value ε_d_P* for ε_d_ selected from the interval (0, 1). The operation of the subtractive potential method is described by the following algorithm:

1. Select r_a_, r_b_, ε_u_ and ε_d_.
2. Determine the values of the potential function P(i) for all points of the set (i = 1, …, N).
3. Choose the point x_u_ with the highest potential P_u_ = P* and assume that it is the first center of the c_1_ cluster.
4. Take k = 2.
5. Loop through the following steps:
  (a) Choose the point x_u_ with the highest P_u_ potential.
  (b) If P_u_ > ε_u_P* then xu becomes the center of the k-th cluster. If ε_u_P* > P_u_ > ε_d_P* then x_u_ becomes the center of the k-th cluster c_k_ if it meets additional conditions (depending on the algorithm implementation method).
  (c) Take k = k + 1.
  (d) If P_u_ > ε_d_P* exit the loop—there are no more cluster centers.

The center of the cluster found in the training data are the points in the feature space whose neighborhood is mapped to a given class. Each cluster center can be translated into a fuzzy class identification rule. The generalized type-1 TSK model can be described by fuzzy IF-THEN rules that represent the input-output relationships of the system. For a first-order multi-input, single-output (MISO) type-1 TSK model, the k-th rule can be expressed as:

(8) $$\mathrm{IF}\;x_{1}\;\mathrm{is}\;Q_{1k}\;\mathrm{and}\;x_{2}\;\mathrm{is}\;Q_{2k}\;\mathrm{and}\;\ldots \;\mathrm{and}\;x_{n}\;\mathrm{is}\;Q_{nk},\;\mathrm{THEN}\;Z\;\mathrm{is}\;w^{k}=p_{0}^{k}+p_{1}^{k}x_{1}+p_{2}^{k}x_{2}+\cdots +p_{n}^{k}x_{n}$$

where x_1_, x_2_, …, x_n_ and Z are linguistic variables; Q_1k_, Q_2k_, …, Q_nk_ are fuzzy sets; X_1_, X_2_, …, X_n_, and $p_{0}^{k},p_{1}^{k},p_{2}^{k},\ldots ,p_{n}^{k}$ are regression parameters.

In the subtractive grouping method, x_j_ is the j-th input feature of x_j_ (j ∈ [1, n]), and Q_jk_ is the MF in the k-th rule associated with the j-th input feature. MF Q_jk_ can be obtained as:

(9) $$Q_{jk}=exp\left[-\frac{1}{2}{\left(\frac{x_{j}-x_{jk}^{*}}{\sigma }\right)}^{2}\right]$$

where x_jk_^*^ is j-th input feature, σ is the Gaussian standard deviation MF given as:

(10) $$\sigma =\sqrt{1/2\alpha }$$

**Figure 13:**
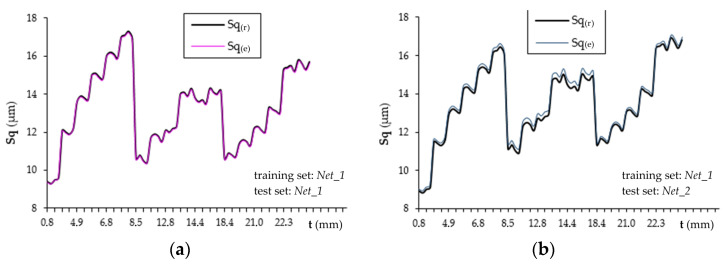
Evaluation of the surface roughness using the TSK fuzzy system: (**a**) training set *Net_1*, test set *Net_1*, (**b**) training set *Net_1*, test set *Net_2*.

Using the TSK fuzzy approach, it is possible to obtain a TSK fuzzy model with rules describing the Sq and selected measurements of the acceleration signals and kinematic parameters as input variables. System identification using clustering involves creating clusters in the data space and translating these clusters into TSK rules so that the model obtained is similar to the identified system. The cluster radius is limited to the range [0.15; 1.0] in steps of 0.15. Figure 13 summarizes the Sq prediction results from the *Net_1* (training) set and the *Net_2* (testing) set. The results of the presented method fit the experimental data better than the results obtained using the RBF neural network.

Table 5 compares the error of estimating the value of the Sq parameter obtained using the RBF network and fuzzy logic. TSK has the lowest root mean square error.

## 4. Conclusions

From the analysis of the presented results, the RBF neural network used in the research as well as the TSK fuzzy logic are very well suited for the automatic assessment of surface roughness based on the used measurements of acceleration signals and kinematic parameters of the MDF milling process. In terms of implementation, these methods are the easiest tools for mapping measurements and cutting parameters in multidimensional space. A neural network does not require special attention to input data. It is enough to determine the signal measurements and the structure of the network and then train the network to get the expected mapping.

When creating a base of inference rules for a fuzzy system, it is necessary to carefully analyze the waveforms of signal measurements. The rules of a fuzzy system are simple, and their construction consists in transferring, almost ‘directly’, the observation of the changes in measurements to the language of fuzzy logic. Both RBF networks and fuzzy system respond well to input variables. With the help of two tested artificial intelligence tools, it is possible to estimate the surface roughness of the workpiece with a small error. When using an RBF neural network, the RMSE of estimating the value of the Sq parameter was 0.379 μm, while the estimation error based on fuzzy logic was 0.198 μm. The results of the experiments show the effectiveness of fuzzy logic and a satisfactory comparison with other methods of artificial intelligence.

The error in the evaluation of the Sq parameter results not so much from the imperfection of the mapping tools, but from the dispersion of the measurements, mainly caused by errors in measuring the value of the Sq parameter and the non-uniformity of the workpiece material. The advantage of an RBF network is its training time—much shorter than for training a multilayered perceptron ANN. In the future, research is planned that will be extended to other types of networks, that is, a self-learned ANN and a neuro-fuzzy system.

## Figures and Tables

**Table 1:** Selected mechanical and physical properties of the MDF.

Density (kg/m^3^)	Moisture Content (%)	Bending Strength (MPa)	Elasticity Modulus (Mpa)	Thermal Conductivity (W/m·K)	Thermal Expansion (µm/m·K)
742	7.2	38	2530	0.3	12

**Table 2:** Geometric parameters of the cutting toll (α—clearance angle, γ—rake angle).

D (mm)	B (mm)	L (mm)	d (mm)	α (×)	γ (°)
12	51	108	12	20	7

**Table 3:** The machining parameters.

Cutting Speed v_c_ (m/min)	Feed per Tooth f_z_ (mm)	Feed Rate v_f_ (mm/min)	Tool Rotational Speed v_c_ (rpm)	Depth of Cut (mm)	Width of Cut (mm)
38	0.30	100	1000	6	5
	0.25	200			
	0.20	300			
	0.15	400			
	0.10	500			
	0.05	600			
76	0.30	200	2000	6	5
	0.25	400			
	0.20	600			
	0.15	800			
	0.10	1000			
	0.05	1200			
114	0.30	300	3000	6	5
	0.25	600			
	0.20	900			
	0.15	1200			
	0.10	1500			
	0.05	1800			

**Table 4:** Comparison of network predicted RMSEs depending on the adopted signal measurement.

Signal Feature	RMSE (μm)									
	0.377	0.347	0.242	0.287	0.248	0.334	0.223	0.231	0.289	0.209
Maximum	x									
Standard deviation	x	x	x							
Root mean square	x	x	x	x	x	x	x	x		x
Skewness	x	x	x	x	x	x	x	x	x	x
Kurtosis	x	x	x	x	x	x	x			
Energy	x	x	x	x	x					
Shannon entropy	x	x	x	x	x	x	x	x	x	x
Log energy entropy	x	x	x	x	x	x				
4th moment	x	x	x	x						
Impulse	x	x								
Cutting speed	x	x	x	x	x	x	x	x	x	x
Feed rate	x	x	x	x	x	x	x	x	x	x

**Table 5:** Summary of RMSEs obtained by different methods of artificial intelligence.

Artificial Intelligence Methods	Training Set: *Net_1*	Test Set: *Net_2*
	RMSE (μm)	RMSE (μm)
RBF neural network	0.273	0.379
TSK	0.066	0.198
